# ESC Congress 2015, London: hot lines truly hot?

**DOI:** 10.1007/s12471-015-0750-1

**Published:** 2015-09-25

**Authors:** E.E. van der Wall

**Affiliations:** Netherlands Society of Cardiology, Holland Heart House, Moreelsepark 1, 3511 EP Utrecht, The Netherlands

At the ESC Congress 2015 in London, six different Hot Line sessions were organised. The six topics were acute myocardial infarction, atrial fibrillation/pacing, diabetes/pharmacology, hypertension, heart failure, and coronary artery disease. Hot Line sessions are usually the main attraction of the ESC Congress as novel data are being presented for the first time [1]. Altogether 26 novel studies were presented during the six Hot Line sessions. All these Hot Line studies will be reported in detail in a special NHJ supplement that is added to this November edition of our journal. Therefore, the outcomes of all these trials will not be addressed here.

The focus of this Comment is to briefly highlight those trials that had a negative (or neutral) outcome. Over the past years, trials with a negative outcome seem to outnumber trials with a positive outcome during the annual ESC Congresses [2, 3]. The following studies mentioned below had a negative outcome for their primary endpoints at the ESC Congress 2015 in London.

1. The CIRCUS (Does **C**yclosporine **I**mp**r**ove **C**linical O**u**tcome in **S**T-elevation Myocardial Infarction Patients?) trial failed to show that cyclosporine improved clinical outcomes or prevented adverse left ventricular remodelling in anterior ST-segment elevation myocardial infarction (STEMI). The goal of the trial was to evaluate treatment with cyclosporine compared with placebo among subjects undergoing primary percutaneous coronary intervention for anterior STEMI.
2. The ALBATROSS (**A**ldosterone **L**ethal Effects **B**lockade in **A**cute Myocardial Infarction **T**reated With or Without **R**eperfusion to Improve **O**utcome and **S**urvival at **S**ix Months Follow-up) trial failed to show that aldosterone antagonists were beneficial to patients with myocardial infarction without heart failure.
3. The PRomPT (**P**ost-Myocardial Infarction **R**emodelling **P**revention **T**herapy) trial showed that peri-infarct pacing did not prevent left ventricular remodelling or improve functional, or clinical outcomes during 18 months of follow-up in patients with a large first myocardial infarction.
4. The OPTIDUAL (**Opti**mal Duration of **Dual** Antiplatelet Therapy After Drug-eluting Stent Implantation) trial aimed to evaluate dual antiplatelet therapy (DAPT) for an additional 36 months among subjects who received a drug-eluting stent and were event-free at 12 months. The trial failed to reach primary outcome; however, extended duration of DAPT non-significantly reduced ischaemic events without increasing bleeding.
5. The ARTS-HF (Miner**a**locorticoid **R**eceptor An**T**agonist **S**tudy In **H**eart **F**ailure) trial failed to show a reduction in NT-proBNP levels for finerenone versus eplerenone; however, secondary outcomes favoured finerenone.
6. The TECOS (**T**rial **E**valuating **C**ardiovascular **O**utcomes with **S**itagliptin) trial showed that sitagliptin improves glycaemic control without increasing adverse cardiovascular events. Among diabetic subjects with documented cardiovascular disease, sitagliptin was non-inferior to placebo on the outcome of adverse cardiovascular events.
7. The ELIXA (**E**valuation of **Lix**isenatide in **A**cute Coronary Syndrome) study evaluated treatment with lixisenatide versus placebo in patients with type 2 diabetes presenting with an acute coronary syndrome. Although lixisenatide showed significant changes in biomarkers, a neutral effect upon cardiovascular death, myocardial infarction and stroke was found.
8. The SCOT (**S**tandard Care versus **C**elecoxib **O**utcome **T**rial) showed that adverse cardiovascular event rates were similar between celecoxib and nonselective nonsteroidal anti-inflammatory drugs (NSAID). The goal of the trial was to evaluate treatment with celecoxib compared with a nonselective NSAID among subjects with arthritis and no known cardiovascular disease.
9. The MATRIX (**M**inimizing **A**dverse Hemorrhagic Events by **T**rans**r**adial Access Site and Systemic **I**mplementation of Angio**X**) trial showed that bivalirudin did not reduce major or net adverse events versus heparin; however, bleeding/death was reduced and stent thrombosis increased. Several lines of evidence even suggest an increased risk of stent thrombosis with bivalirudin.
10. The BENEFIT (**BEN**znidazole **E**valuation **F**or **I**nterrupting **T**rypanosomiasis) study showed that benznidazole did not reduce progression of Chagas disease cardiomyopathy among patients with Chagas disease. However, it was found that a 40–80 day treatment with this antiparasitic medication significantly reduced parasitic activity in the blood.
11. The UNDER-ATP (Atrial Fibrillation/Pacing Adenosine Triphosphate) trial showed that guided pulmonary vein isolation in comparison to conventional pulmonary vein isolation did not reduce late recurrence atrial fibrillation at 1 year follow-up.
12. The PRESERVATION I showed that a novel Bioabsorbable Cardiac Matrix (BCM) is safe but does not benefit STEMI patients with large infarcts; it does not prevent left ventricular remodelling or other adverse outcomes.
13. The ‘Optimization of Heart Failure Management Using Medtronic **Opti**Vol Fluid Status Monitoring and Care**Link** Network’ (OptiLink) study assessed the effect of intrathoracic impedance monitoring, through automatic wireless telemedicine notification on all-cause death and cardiovascular hospitalization. The primary composite endpoint did not differ between the two groups (telemedicine guided system or controls).
14. The ‘**C**alcium **U**p-Regulation by **P**ercutaneous Administration of Gene Therapy **i**n Cardiac **D**isease Phase 2b’ (CUPID2) trial, investigating the efficacy and safety of intracoronary administration of adeno-associated virus type I (AAV1)/SERCA2a in patients with advanced heart failure, showed that treatment with AAV1/SERCA2a was safe but failed to improve the rate of recurrent events as well as the time to the first terminal event.
15. The ‘Treatment of Sleep-Disordered Breathing with Predominant Central Apnoea with Adaptive **Ser**vo-**Ve**ntilation in Patients with Chronic **H**eart **F**ailure’ (SERVE-HF) showed that the composite primary endpoint (time to first event of all-cause death, life-saving intervention, or unplanned hospitalization for chronic heart failure) did not differ between intervention and control group. In fact, all-cause death and cardiovascular death were elevated in the treatment arm.

At a first glance the ‘negative’ outcomes of these trials seem rather disappointing. One could therefore really wonder whether all Hot Line studies were truly hot. However, it should be realised that the label ‘positive’ or ‘negative’ hinges on a p-value of < 0.05 or > 0.05; this might in principle have nothing to do with the clinical implication of a study, be it positive or negative. Secondly, ‘negative’ usually only applies to the primary endpoint; in some instances the drugs studied in the above-mentioned trials showed improved safety beyond the neutral finding. Finally, the ultimate proof of the value of a large clinical study is incorporation into the guidelines [4–8].

Nevertheless, there is a flipside of the coin. Sometimes one cannot escape the feeling that also in science there is a *l’art pour l’art* approach; are all the above-described studies really needed? Are the studies truly hypothesis-driven? Will the outcome of the study change the treatment policy for the individual patient? Finally, will it impact the existing guidelines [9, 10]? Therefore, the impression occasionally arises that some studies are primarily performed for the sake of the industry, followed by the urge of the researchers, and lastly in the interest of the patient. I sincerely hope this impression is false.
